# Electroacupuncture Pretreatment Alleviates Cerebral Ischemia-Reperfusion Injury by Increasing GSK-3*β* Phosphorylation Level via Adenosine A1 Receptor

**DOI:** 10.1155/2020/6848450

**Published:** 2020-02-20

**Authors:** Wujun Geng, Libin Cai, Kunyuan Han, Ding Li, Yunchang Mo, Qinxue Dai, Hongli Tang, Minyuan Zhang, Percy David Papa Akuetteh, Meita Felicia Balelang, Junlu Wang

**Affiliations:** ^1^Department of Anesthesiology, The First Affiliated Hospital of Wenzhou Medical University, Wenzhou, Zhejiang, China; ^2^Department of Anesthesiology, The Yongjia Hospital of TCM, Wenzhou, Zhejiang, China; ^3^Department of Anesthesiology, The People's Hospital of Wencheng County, Wenzhou, Zhejiang, China

## Abstract

**Objective:**

To observe the effect of adenosine A1 receptor in the hippocampus of mice on GSK-3*β* phosphorylation level and elucidate the underlying mechanisms of electroacupuncture pretreatment by activating Α1 receptor mediating cerebral ischemia-reperfusion injury.

**Method:**

The model of middle cerebral artery occlusion (MCAO) was established and grouped into electroacupuncture pretreatment group (EA group), MCAO group, and sham-operated group (Sham group). The neurobehavioral manifestation, the volume of cerebral infarction, and its related protein changes in mice in each group were observed. Then, adenosine Α1 receptor antagonist and agonist were injected intraperitoneally to observe the effects of A1 receptor on the phosphorylation level of GSK-3*β* phosphorylation level and elucidate the underlying mechanisms of electroacupuncture pretreatment by activating Α1 receptor mediating cerebral ischemia-reperfusion injury.

**Results:**

(1) Compared with the MCAO group (24 hours after reperfusion), the infarct size in the EA group decreased significantly, and the Garcia neurological score and phosphorylation level of GSK-3*β* phosphorylation level and elucidate the underlying mechanisms of electroacupuncture pretreatment by activating Α1 receptor mediating cerebral ischemia-reperfusion injury. *β* phosphorylation level and elucidate the underlying mechanisms of electroacupuncture pretreatment by activating Α1 receptor mediating cerebral ischemia-reperfusion injury. *β* phosphorylation level and elucidate the underlying mechanisms of electroacupuncture pretreatment by activating Α1 receptor mediating cerebral ischemia-reperfusion injury.

**Conclusions:**

Electroacupuncture pretreatment can increase GSK-3*β* phosphorylation level via activating A1 receptor, to protect neurons in ischemia-reperfusion injury.*β* phosphorylation level and elucidate the underlying mechanisms of electroacupuncture pretreatment by activating Α1 receptor mediating cerebral ischemia-reperfusion injury.

## 1. Introduction

Ischemic stroke is a common disease of the nervous system. It is a clinical problem with a high mortality and disability rate. The disease leads to physical disabilities and severe cognitive impairment, which mostly needs urgent medical treatment. The risk of cerebral ischemia caused by vasospasm also exists in cerebrovascular operations such as aneurysm clipping and intervention [1]. Once this risk occurs, the perfusion is severely reduced and the cells begin to swell and necrose. The perfusion should be restored within a limited time window to avoid further deterioration of the neural function [2]. Restricted by the narrow therapeutic time window, the available clinical interventions are very limited. Hence, the starting point of our research is to prolong the time of nerve cell tolerance in ischemia.

An adequate assessment must be made before the patient undergoes cerebrovascular surgery [1]. It is important to slow down the ATP consumption to enhance the tolerance of ischemic tissue through positive pretreatment [3]. As the development of traditional Chinese medical technology “acupuncture,” electroacupuncture has been proved effective in analgesia and protection of transient ischemic attack [4, 5]. In previous studies, we found that electroacupuncture pretreatment enhanced the ischemic tolerance of neurons in the hypoperfusion area, which was induced by the activation of adenosine A1 receptor [6].

CCPA is an A1 receptor agonist, and it was found to promote phosphorylation of GSK-3*β* in a screening trial of protective drugs for myocardial ischemic tolerance [7]. Similarly, in a liver ischemic tolerance test that was mediated by the Α1 receptor, CCPA caused a significant increase in P-GSK-3*β* [8]. GSK-3*β* is the convergence point of the cellular protective signaling pathway, which plays an important role in the regulation of neuronal polarity, neuroinflammation, and neurodegenerative disorders [9, 10]. GSK-3*β* also mediates the survival of vulnerable hippocampal CA1 neurons after transient global cerebral ischemia (tGCI) [11].

In our trial test, the expression of GSK-3*β* in the hippocampus of mice was correlated with the activity of adenosine A1 receptor. The upregulation of phosphorylated GSK-3*β* after EA treatment is consistent with the conclusion of Wei et al. on Sprague Dawley rats [5]. To clarify this possible mechanism, a mouse model of middle cerebral artery occlusion (MCAO) was constructed and then treated with electroacupuncture or A1 receptor agonists/antagonists to explore the relationship between adenosine A1 receptor and P-GSK-3*β*, which may lead to the protective effect of electroacupuncture.

## 2. Materials and Methods

### 2.1. Animals and Drugs

Male C57BL/6 mice were purchased from Beijing Vital River Laboratory Animal Technology Co., Ltd. All animal procedures were approved by the Ethics Committee of Animal Experimentation and received humane care in accordance with the guidelines for Animal Experiments of Wenzhou Medical University. The mice were housed under 12-hour light and dark cycles at a temperature of 20–25°C and the air humidity was at 60%. Water and food are freely available. The mice weighed 20–25 g and were randomly divided into different treatment groups.

2-Chloro-N^6^-cyclopentyladenosine (CCPA), a highly selective adenosine A1 receptor agonist, was injected intraperitoneally (i.p.) at a dose of 0.2 *μ*g/g in 5% dimethyl sulfoxide (DMSO) [12–15], 30 minutes before MCAO. 8-Cyclopentyl-1,3-dipropylxanthine (DPCPX), a highly selective adenosine A1 receptor antagonist, was injected at a dose of 1 *μ*g/g (i.p.) in 5% DMSO [12–15], thirty minutes before EA. Lithium chloride (LiCl), the GSK-3*β* inhibitor, which can phosphorylate the GSK-3*β* into P-GSK-3*β*, was injected at a dose of 90 *μ*g/g (i.p.) in physiological saline [16], 30 minutes before MCAO. The above drugs were purchased from Sigma-Aldrich (St. Louis, MO, USA). Wortmannin (Wrt), a selective phosphatidylinositol 3-kinase (PI3K) inhibitor, purchased from Beyotime (Beijing, CHN), was injected at a dose of 1 *μ*g/g (i.p.) in 5% DMSO [17], 30 minutes before MCAO.

### 2.2. Middle Cerebral Artery Occlusion Model

In order to construct the middle cerebral artery occlusion (MCAO) in mice, we referred to the improvements and summaries proposed by Shimamura in 2006 [18]. Α silicon-coated monofilament nylon suture obtained from RWD Life Science (Shenzhen, Guangzhou, CHN) was passed through the external carotid artery, bifurcation of the common carotid artery, and internal carotid artery into the origin of middle cerebral artery, which resulted in ischemia of the middle cerebral artery blood supply area. The silicon-coated monofilament nylon suture was fixed in place for 90 minutes and removed, and reperfusion was done via the common carotid artery [19].

### 2.3. Electroacupuncture Pretreatment

Mice were anesthetized with chloral hydrate (4%, 0.01 mL/g, i.p.) before EA pretreatment. According to experimentally based acupoint selection principles [20], the *Baihui* acupoint is located at the intersection of the line linking the two mouse ear tips and the sagittal midline. To construct a circuit, we chose the *Baihui* acupoint as one electrode site and the Shenting (the nonacupoint point) as the other. Needle depth was 1 mm and it was taped. We used a HAN's EA Instrument (HANS, Beijing, CHN) to stimulate the *Baihui* acupoint for 30 minutes at a frequency of 2/15 Hz and an intensity of 1 mA.

### 2.4. Neurological Deficit Evaluation

We measured neurological scores 24 h after cerebral ischemia-reperfusion. We assessed mice's sports coordination ability with a modified Garcia's neurological scoring system including (Table 1): (1) spontaneous activity, (2) symmetry of movement, (3) floor walking, (4) beam walking, and (5) response to vibrissae touch. Each test was assigned a score from 0 to 3. The minimum neurological score is 1 and the maximum is 15 [21, 22]. The adhesive removal test also called sensory asymmetry test was used to determine the skin sensitivity and the integration of sensory function in mice. The specific method is to attach 6 mm square back glue label to the relatively hairless ankle of mice, when returned to the cage, a normal mouse quickly removed each square label with its teeth. In mice with cerebral ischemia, the label attached to the normal hind limb was removed first and then the lesion side, which took more time because ischemic lesions lead to sensory asymmetry. In addition, delays in time also indicate a lack of motor function, and movement disorders in the mouth and hind limbs lead to relatively difficult removal of sticky labels [23].

### 2.5. Measurement of Infarct Size

24 hours after reperfusion, the animals were euthanized with excessive chloral hydrate, the brains were removed quickly, and 2 mm thick coronal sections were made from anterior to posterior. The sections were dyed at 37°C for 20 minutes with 2% 2,3,5-triphenyltetrazolium chloride (TTC) solution and then fixed with 4% paraformaldehyde for 24 hours. The pictures were taken, scanned, and then analyzed with Image-Pro Plus 6.0 software system. After correction of swelling, the relative infarct area was measured according to the following equation: relative infarct size = (contralateral area − ipsilateral noninfarct area)/contralateral area [24].

### 2.6. Western Blot

24 hours after reperfusion, the animals were euthanized with excessive chloral hydrate, and the hippocampus was rapidly separated on infarcted side. The operation should be performed on a cold surface, and liquid nitrogen was used to terminate the cell metabolism. The tissues were homogenized in 100 RIPA lysis buffer (Beyotime, CHN): 10 phosphatase inhibitors (Roche, Germany): 1 PMSF (Beyotime, CHN) on ice. Tissue extract was centrifuged at 12000*g* at 4°C for 30 min. Western blotting was performed with standard procedures. PVDF membranes were incubated with GSK-3*β* polyclonal antibody, phospho-GSK-3*β* (Ser-9) polyclonal antibody (1 : 1000 dilution, Affinity Biosciences, USA), adenosine A1 receptor polyclonal antibody (1 : 1000 dilution, Abcam, USA), and GAPDH polyclonal antibody (1 : 1000 dilution, Hangzhou Xianzhi, CHN) at 4°C for 16 hours. After that, the membranes were incubated for 1 hour with the horseradish peroxidase- (HRP-) conjugated goat anti-rabbit (1 : 5000 dilution, Biosharp, CHN) at room temperature.

### 2.7. Immunohistochemistry

24 hours after reperfusion, the animals were euthanized with excessive chloral hydrate and perfused with 4% cold paraformaldehyde, and the brain was fixed with 4% paraformaldehyde at 4°C overnight. After paraffin embedding, reference to the Anatomical Map of Mouse Brain, slices with a thickness of 5 microns were made along the coronal plane [25]. Then sodium citrate was used for antigen repair, and 3% hydrogen peroxide was used to inactivate endogenous peroxidase at room temperature for 30 minutes. After blocked with 3% BSA at room temperature for 1 hour, the tissue slices were incubated with primary antibody which dissolved in 3% BSA overnight at 4°C. Then, the secondary antibody was used to incubate the tissue slices at the room temperature for 1 hour (the antibodies in immunohistochemistry and WB were identical, the primary antibody concentration was 1 : 100, and the secondary antibody concentration was 1 : 400). DAB staining and hematoxylin staining were used with staining rack to make sure all sections had the same reaction time. According to the different pretreatment methods, five groups were divided, each group had four samples, each sample was sliced at a thickness of 5 *μ*m, and two consecutive pieces were selected for observation and image capturing, and the relative grey density of hippocampal CA1 area under 400 times microscope was taken.

Images of the immunostaining were digitally captured by using a Leica ICC 50 W microscope. The further processing of the images was treated by Image-Pro Plus 6.0 (IPP6.0, Media Cybernetics, USA).

### 2.8. Statistical Analysis

Except the infarct size using percentage, other values are represented as mean ± SEM. GraphPad Prism 7.0 (GraphPad, San Diego, CA, USA) was used in statistical analysis. Normal distribution test was conducted by Kolmogorov–Smirnov test. The date of infarct size, western blot, and adhesive removal test were performed using one-way ANOVA, followed by Bonferroni correction for the post hoc T test. Wilcoxon signed-rank test (Wilcox test) was used to compare neurological deficit scores. *P* < 0.05 was accepted as statistically significant.

## 3. Result

**Figure 1:**
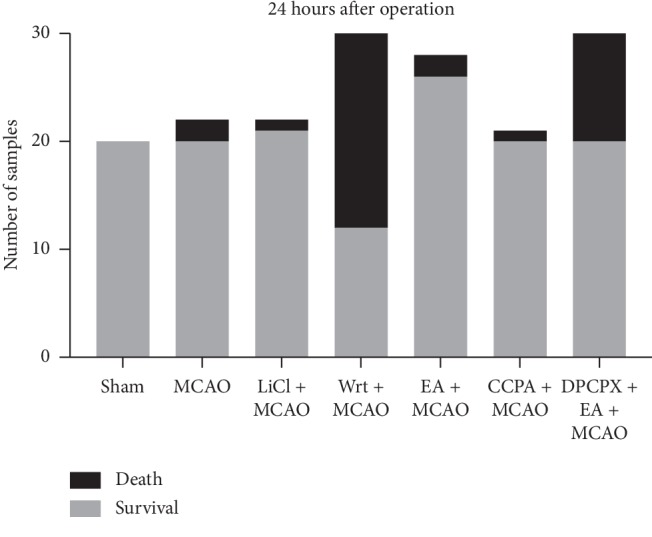
The experimental factors significantly affected the survival of the samples.

We used a nylon embolus to make an ischemia-reperfusion model. The validity of the models for each group was determined by TTC staining. All sham-operated mice survived 24 hours after the operation (*n* = 20). The mortality rate among the MCAO group (*n* = 22), LiCl + MCAO group (*n* = 22), EA + MCAO group (*n* = 28), and CCPA + MCAO group (*n* = 21) showed no significant difference, and it was about 5–10%, but the DPCPX + EA + MCAO group reached 33% (*n* = 30) and the Wrt + MCAO group reached 60% (*n* = 30) (Figure 1).

### 3.1. GSK-3*β* Phosphorylation May Be a Key Factor Affecting Cerebral Ischemia-Reperfusion Injury

**Figure 2:**
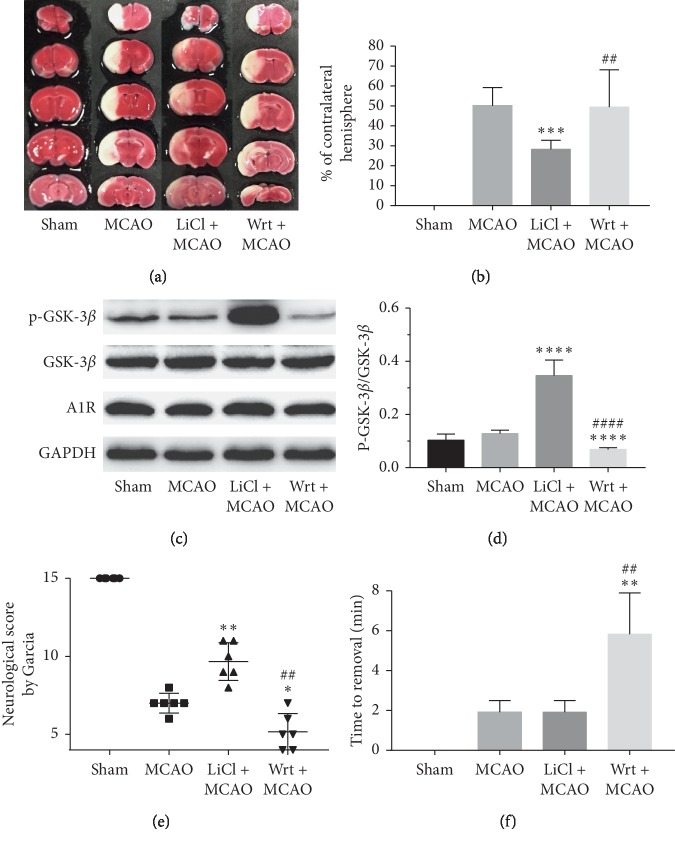
(a) TTC staining of cerebral infarction. (b) Infarct size. (c, d) Western blot analysis. (e) Modified Garcia's neurological score. (f) Adhesive removal test.

The drug lithium chloride, which promotes the phosphorylation of GSK-3*β*, and the drug Wortmannin, which inhibits the phosphorylation of GSK-3*β*, were injected 30 minutes before the establishment of the model. TTC staining, western blot, and neurological deficit evaluation were performed 24 hours after reperfusion. Compared with the MCAO group, the infarct size in the LiCl + MCAO group decreased significantly (*P* < 0.001, Figures 2(a) and 2(b)), the phosphorylation level of GSK-3*β* was markedly increased (*P* < 0.0001, Figures 2(c) and 2(d)), together with higher modified Garcia's neurological score (*P* < 0.001, Figure 2(e)), but the adhesive removal test showed no significant difference between the two groups. Compared with the MCAO group, the infarct size in the Wrt + MCAO group tended to increase, but there was no significant difference between the Wrt + MCAO group and the MCAO group. The possible explanation is that the further expansion of the injured area will affect the survival of mice after reperfusion, which is reflected in the high mortality rate of the Wrt + MCAO group. The phosphorylation level of GSK-3*β* in the Wrt + MCAO group was also significantly lower than that in the MCAO group (*P* < 0.0001, Figures 2(c) and 2(d)), as well as the modified Garcia's neurological score (*P* < 0.05, Figure 2(e)). The adhesive removal test showed significant sensory impairment in hind limbs (*P* < 0.01, Figure 2(f)).

### 3.2. The Effect of Adenosine A1 Receptor on Phosphorylation of GSK-3*β* Is an Important Mechanism for Brain Protection Induced by Electroacupuncture Pretreatment

#### 3.2.1. Electroacupuncture Pretreatment Alleviates Cerebral Ischemia-Reperfusion Injury While Increasing GSK-3*β* Phosphorylation Level

**Figure 3:**
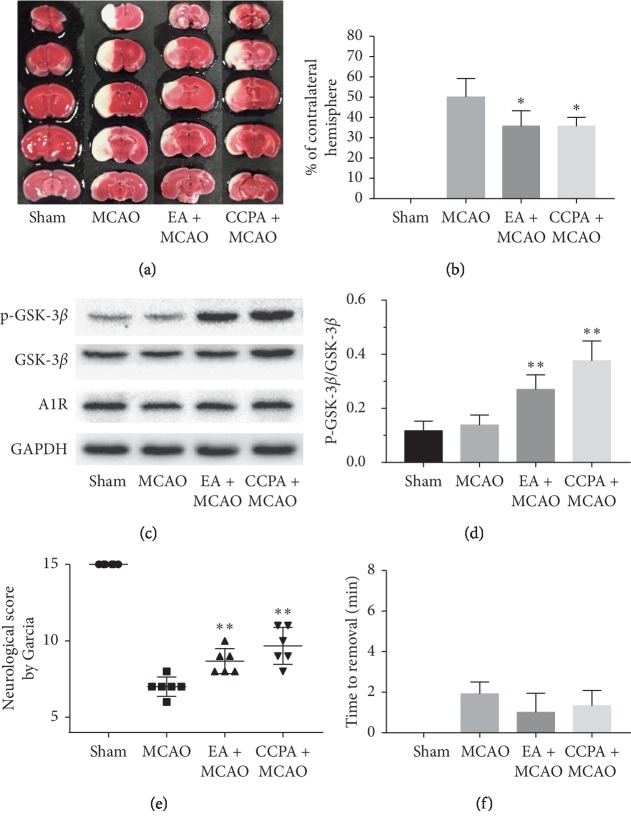
(a) TTC staining of cerebral infarction. (b) Infarct size. (c, d) Western blot analysis. (e) Modified Garcia's neurological score. (f) Adhesive removal test.

**Figure 4:**
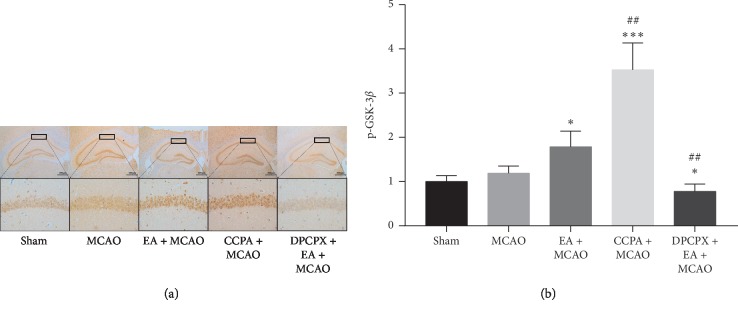
(a, b) Immunohistochemical analysis.

We performed electroacupuncture pretreatment for 30 minutes and left middle cerebral artery embolization two hours later. Compared with the MCAO group, the EA pretreatment group performed better in subsequent cerebral ischemia-reperfusion injury, with smaller infarct area and a considerable number of nerve cells in the penumbra survived (*P* < 0.05, Figures 3(a) and 3(b)). Correspondingly, the EA + MCAO group performed better than the MCAO group in cerebral ischemia-reperfusion injury to motor function (*P* < 0.01, Figure 3(e)). Although the EA + MCAO group tended to reduce sensory impairment, there was no significant statistical difference (*P* > 0.05, Figure 3(f)). There was no difference in P-GSK-3*β*/GSK-3*β* between the MCAO group and the Sham group in western blotting. P-GSK-3*β*/GSK-3*β* in the EA + MCAO group was significantly higher than that in the MCAO group (*P* < 0.01, Figures 3(c) and 3(d)). Similarly, P-GSK-3*β*/GSK-3*β* in the EA + MCAO group was also higher than that in the MCAO group in the immunohistochemical experiment of CA1 area in the hippocampus of mice (*P* < 0.05, Figures 4(a) and 4(b)).

#### 3.2.2. The Activation of Adenosine A1 Receptor Increased P-GSK-3*β*, Mimicking the Protective Effect of Electroacupuncture on Brain

We injected CCPA (i.p.) 30 minutes before the MCAO. And we observed a significant reduction in infarct size in this group (*P* < 0.05, Figures 3(a) and 3(b)). Correspondingly, the injury of motor function in the CCPA + MCAO group was slighter than that in the MCAO group (*P* < 0.01, Figure 3(e)), and there was no statistical difference between the CCPA + MCAO group and EA + MCAO group. Although we observed that the infarct size and motor function of CCPA + MCAO group improved significantly compared with the MCAO group, but there was no difference in sensory impairment test. The exciting thing is that activation of adenosine A1 receptor increased the phosphorylation level of GSK-3*β*. We obtained the same results in western blot (*P* < 0.01, Figures 3(c) and 3(d)) and immunohistochemical experiments (*P* < 0.001, Figures 4(a) and 4(b)). Therefore, we hypothesized that electroacupuncture could increase P-GSK-3*β* by activating adenosine A1 receptor, thus producing brain protection. To test this hypothesis, we used DPCPX, a selective inhibitor of adenosine A1 receptor, before electroacupuncture treatment.

#### 3.2.3. DPCPX, a Selective Inhibitor of Adenosine A1, Reduced P-GSK-3*β* and Reversed the Protective Effect of Electroacupuncture on Brain

**Figure 5:**
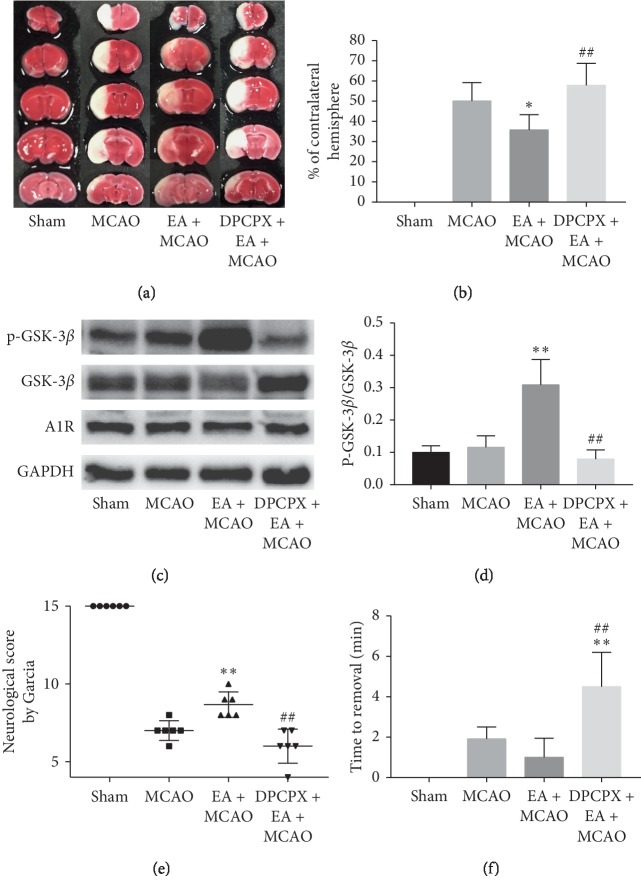


We injected selective adenosine A1 inhibitor DPCPX 30 minutes before electroacupuncture treatment. Then, the ischemia-reperfusion model was established by following the project protocol. The use of DPCPX reversed the effect of electroacupuncture on reducing infarct size (*P* < 0.01, Figures 5(a) and 5(b)). Correspondingly, the motor and sensory impairments in the DPCPX + EA + MCAO group were significantly worse than those in the EA + MCAO group (*P* < 0.01, Figures 5(e) and 5(f)). There was no significant difference in infarct size and motor function damage between DPCPX + EA + MCAO group and MCAO group, but sensory impairment was aggravated (*P* < 0.01, Figure 5(f)). In western blotting, DPCPX reversed the effect of EA on P-GSK-3*β* elevation (*P* < 0.01, Figure 5(d)). There was no significant difference in GSK phosphorylation between DPCPX group and MCAO group. The same result was also found in immunohistochemistry of CA1 region in the hippocampus of mice (*P* < 0.01, Figures 4(a) and 4(b)). These results suggest that electroacupuncture induced GSK-3*β* phosphorylation which is adenosine A1 receptor dependent.

## 4. Discussion

Although ischemic preconditioning enhances the tolerance of ischemic tissues and has been widely accepted for its protective effect on cerebral ischemia, this artificial method of transient ischemia has high risk and complex operation, which limits its clinical application [26]. As the development of traditional Chinese acupuncture and moxibustion, electroacupuncture has the characteristics of simple operation, safety, and reliability. Electroacupuncture preconditioning plays a similar role in ischemic preconditioning in brain protection. We intend to prove that electroacupuncture pretreatment by excited adenosine Α1 receptor induced phosphorylation of GSK-3*β*, thus improving the tolerance of nerve cells to ischemia.

Adenosine A1 receptor receives energy metabolite adenine nucleoside, which plays a feedback role in the body's metabolic regulation. It also acts on vascular endothelium, expanding blood vessels and increasing blood supply. In this study, significant phosphorylation of GSK-3*β* was observed in the application of adenosine A1 receptor agonist.

GSK-3*β* is a versatile Ser/Thr kinase that phosphorylates more than 20 substrates [27]. The role of P-GSK-3*β* in cerebral protection during ischemia-reperfusion has been confirmed. The possible reasons may be that P-GSK-3*β* reduces the sensitivity of neuronal apoptosis by inhibiting the activation of caspase-3 [28–30] or maintains the transmembrane potential after mitochondrial reperfusion injury by inhibiting the opening of mitochondrial permeability transition pore (mPTP) [17, 31, 32]. There is more evidence that GSK-3*β* affects the activity of pyruvate dehydrogenase, and phosphorylated GSK-3*β* enhances the glycolysis ability and glucose utilization of nerve cells [33–35].

In the first experiment, we used drugs to increase or decrease P-GSK-3*β*, western blot was used to test the efficacy of these drugs, TTC staining was used to measure the infarct volume, and modified Garcia's score and sticker test were used to judge the degree of motor and sensory impairment. We observed that elevated GSK-3*β* phosphorylation significantly reduced infarct size and motor function impairment. Conversely, reduction in the phosphorylation level of GSK-3*β* caused the expansion of infarct volume and aggravated the impairment of motor and sensory function. It is confirmed that P-GSK-3*β* plays a key role in ischemia-reperfusion injury.

In our unpublished studies, we found that adenosine A1 receptor and P-GSK-3*β* were involved in the brain protection of electroacupuncture, so we hypothesized that there might be a link between adenosine A1 receptor and PGSK. This connection between them has not been reported before.

Therefore, in the second group of experiments, we used electroacupuncture pretreatment and adenosine A1 receptor agonist and tested the same indicators. We observed that both of them reduced the infarct area and motor function injury, and it was inspiring to find that P-GSK-3*β* also increased with the use of adenosine A1 receptor agonist. This result is also consistent with our previous hypothesis that the excitation of adenosine A1 will cause an increase of P-GSK-3*β*.

In order to understand whether electroacupuncture increases P-GSK-3*β* through adenosine A1 receptor or it directly acts on GSK-3*β* to produce brain protection, we designed a third group of experiments. The experiment has used adenosine Α1 receptor inhibitor. We found that the addition of adenosine A1 inhibitor completely reversed the cerebral protective effect of electroacupuncture pretreatment, and the phosphorylation of GSK-3*β* was eliminated after electroacupuncture treatment.

In conclusion, it is reasonable to believe that adenosine A1 receptor can regulate P-GSK-3*β*, and this mechanism plays an important role in electroacupuncture alleviating ischemia-reperfusion injury.

In addition, we also noticed that the creeping movement of mice after MCAO was different from that of rats. The main manifestation of hind limb motor function impairment in mice was weakening or loss of adduction, which in SD rats decreased their ability to stand upright on the ground. So, after 90 minutes of ischemia, the mice deflected to the ischemic side when they were moving on the ground. Only when the infarct area was further enlarged, the muscular strength of hind limbs would be further damaged as in rats, that is, to the opposite side of the ischemic focus. At present, there are many literature studies describing the motor function of rats after MCAO, but the motor function of mice after MCAO was rarely described. This discussion gives a relatively detailed description of this difference, which may be explained by differences in the blood supply areas of the middle cerebral artery between mice and rats [23, 36].

## 5. Limitation and Expectation

### 5.1. The Survivorship Bias

The middle cerebral artery ischemia-reperfusion model has a requirement for infarction time [19] and must continue throughout the stable phase of acute infarction, so that the area of acute infarction does not expand, hence controlling the mortality rate of this surgery. After reversing some protective factors artificially, the area of acute infarction becomes more enlarged. This damage will destroy the center that supports basic survival and greatly increase the mortality rate, whether before or after reperfusion. In this experiment, the use of Wortmannin, a drug that inhibits GSK-3*β* phosphorylation, results in extremely high mortality. A large number of death samples were concentrated between 5 and 12 hours after reperfusion, and the infarcted brain tissue was severely edematous. The surviving samples were in infarct size and physiological function. The damage was slightly less than the overall expectation and statistically produced a similar “survivor bias” effect.

### 5.2. Location of Tissue Samples

Due to changes in infarct size, there is no uniform standard for microscopic division of the penumbra in ischemic cerebral cortex. To obtain a relatively stable ischemic tissue, we choose the hippocampus. The hippocampal blood was supplied with the anterior choroidal artery (AchA), the middle hippocampal artery, and the posterior hippocampal artery,and the posterior hippocampal artery. The AchA arises from the internal carotid artery. The middle hippocampal artery and the posterior hippocampal artery arise from the posterior cerebral artery. In the MCAO model, AchA was surgically blocked, but blood flow in the middle and posterior hippocampal arteries was unaffected. The hippocampus presents as stable localized ischemia [37].

## Figures and Tables

**Table 1:** Neurological scoring system, modified from the scoring system developed by Garcia et al.

	Score			
	0	1	2	3
Spontaneous activity (3 min test period)	No movement	Slight movement	Touches 1 or 2 sides of cage	Touches 3 or 4 sides of cage
Symmetry of movement (right forelimb and hind limb)	Total asymmetry	Near-total asymmetry	Mild asymmetry	Complete asymmetry
Floor walking	No walking	Walks in circles only	Curvilinear path	Straight path
Beam walking	Falls off of beam	Hugs beam	Stands on beam	Walks on beam
Response to vibrissae touch of right side		No response	Weak response	Symmetrical response

## Data Availability

All data a available within the manuscript.
